# Associations of Breed and *FUT1* Genotype with the Fecal Microbiota of Weaned Piglets

**DOI:** 10.3390/microorganisms14081756

**Published:** 2026-08-10

**Authors:** Li-Ming Luo, Xu-Yang Lu, Xin-Guo Bao, Zhe-Chen Li, Yue Yang, Wen-Juan Luo, Jing Chen, Wei-Min Lin, Chun-Hui Gao, Rui-Yi Lin

**Affiliations:** 1 College of Animal Sciences, Fujian Agriculture and Forestry University, Fuzhou 350002, China; luoliming@fafu.edu.cn (L.-M.L.); luxuyang@fafu.edu.cn (X.-Y.L.); 52306009010@fafu.edu.cn (X.-G.B.); chloe11097@163.com (Z.-C.L.); woshixiaoyue77@163.com (Y.Y.); lwenjuan@fafu.edu.cn (W.-J.L.); jingc128@163.com (J.C.); weiminlin@fafu.edu.cn (W.-M.L.); 2 National Laboratory of Agricultural Microbiology, Huazhong Agricultural University, Wuhan 430070, China; 3 Key Laboratory of Farmland Remediation in Central China, Ministry of Agriculture and Rural Affairs, Wuhan 430206, China; 4 College of Resources and Environment, Huazhong Agricultural University, Wuhan 430070, China

**Keywords:** weaned piglets, *FUT1*, genotype, 16S rRNA, *Lactobacillus*

## Abstract

This study aimed to analyze the associations between the composition and function of the gut microbiome in weaned piglets of different breeds and Fucosyltransferase 1 (*FUT1*) genotypes. Ear tissue and fecal samples were collected from 163 piglets (61 Durocs, 39 Yorkshires, and 63 Landraces) for genotyping and 16S rRNA sequencing. Three genotypes were identified in the *FUT1* gene: *AA*, *GA*, and *GG*, with the resistant *AA* genotype observed only in Duroc. *α*-diversity analysis revealed that Landrace pigs had the highest microbial diversity, whereas pigs with the *AA* genotype displayed the lowest diversity. At the phylum level, *Bacillota* and *Bacteroidota* were the dominant phyla across all pig breeds and genotypes. At the genus level, significant differences among different groups were observed in microbes such as *Lactobacillus* and *Segatella*. PICRUSt2-based functional prediction revealed a higher relative abundance of predicted functions related to xenobiotics biodegradation and metabolism, digestive system, nucleotide metabolism, and other metabolic processes in the *AA* genotype group. These findings identify breed- and genotype-associated variation in the fecal microbiota of weaned piglets and provide a foundation for future studies investigating whether *FUT1*-associated microbial differences are related to intestinal microbial function and resistance to F18-positive enterotoxigenic *Escherichia coli*.

## 1. Introduction

In intensive livestock production systems, weaned piglets are highly susceptible to diarrhea, edema, and other diseases due to their incompletely developed digestive systems and the stress of weaning [1]. However, traditional technologies such as feeding management, pharmaceutical interventions, and vaccination have gradually reached their limitations in addressing the growing demands for disease prevention and control in modern livestock production. Concurrently, with the rapid advancement of molecular technologies, current research has increasingly focused on identifying disease-resistant genes and selecting genetic markers in pigs.

The Fucosyltransferase 1 (*FUT1*) gene, located on porcine chromosome 6, spans a total length of 1098 base pairs [2]. The porcine *FUT1* c.307G>A missense variant, resulting in a p.Ala103Thr substitution, alters α1,2-fucosyltransferase activity and intestinal mucosal glycosylation [3,4]. Pigs with the *AA* genotype generally exhibit negligible enzyme activity and resistance to F18-fimbriated *Escherichia coli* adhesion, whereas *GA* and *GG* pigs are considered more susceptible. Additionally, studies have shown that pigs carrying the *GA* or *GG* genotype are at increased risk of infections caused by Porcine Reproductive and Respiratory Syndrome Virus (PRRSV) and *Haemophilus parasuis* [5]. These findings suggest that *FUT1* gene polymorphisms may serve as a potential genetic marker for improving the survival of weaned piglets, with pigs bearing the *AA* genotype typically exhibiting higher survival rates [6]. However, the genetic variation in *FUT1* gene polymorphisms varies across pig breeds. For instance, the resistant *AA* genotype is predominantly found in Duroc pigs, whereas Yorkshire and Landrace breeds typically carry the *GA* or *GG* genotypes, and most indigenous Chinese pig breeds exclusively exhibit the susceptible *GG* genotype [7,8]. Although numerous studies have investigated the associations between *FUT1* polymorphisms and traits such as growth performance [9], disease resistance [10], and immune function [11], limited information is available regarding its influence on the gut microbiome composition.

The gut microbiome is essential for host health, forming symbiotic relationships with the digestive and immune systems [12]. Imbalances in the microbiome can alter metabolic gene expression and impair gut function, leading to disorders such as diarrhea in weaned piglets [13]. In practical applications, the composition of the gut microbiome is often modulated by exogenous factors. Research has shown that *xylooligosaccharides* can optimize the gut microbiome structure in pigs, enhance gut barrier function, and regulate immune responses [14]. Additionally, the composition of the gut microbiome is heritable. Studies have identified associations between single nucleotide polymorphisms (SNPs) in certain genomic regions of the porcine genome and the relative abundance of specific microbial genera [15], suggesting that these SNPs may serve as candidate loci for regulating the composition and structure of the gut microbiome.

Given the important roles of host genetic background and the gut microbiome in weaned piglets, the primary aim of this study was to characterize and compare fecal microbial community structure among three commercial pig breeds. The associations between *FUT1* genotype and microbial diversity, community structure, and taxonomic composition, were evaluated as exploratory analyses. These findings provide a basis for future studies examining the biological significance of breed and *FUT1*-associated variation in the fecal microbiota.

## 2. Materials and Methods

### 2.1. Experimental Animals and Samples

A total of 163 piglets representing three breeds (Duroc, *n* = 61; Landrace, *n* = 63; and Yorkshire, *n* = 39) were selected for this study. All animals were obtained from a commercial breeding farm in Fujian Province, China, where they were maintained under standardized feeding regimens and routine disease control protocols. Diets were formulated according to the nutrient requirements of swine (GB/T 39235–2020) [16], and their ingredient composition and nutrient levels are presented in Supplementary Table S1. All sampled piglets belonged to the same contemporaneous farrowing cohort and were offspring of multiparous sows. Sampling covered the offspring of every sow which farrowed in this cohort. Two to three piglets were randomly selected from each litter. The sampling population comprised the offspring of 63 multiparous sows, including 25 Duroc, 15 Yorkshire, and 23 Landrace sows, and all litters produced within the same farrowing cohort were represented. Consequently, the number of piglets included in each breed group was proportional to the number of contributing sows and litters. This sampling strategy was used to reduce the overrepresentation of individual sows and full-sibling families.

Ear tissue samples were collected from newborn piglets, whereas fresh fecal samples were collected after weaning using rectal swabs. At fecal sampling, the average weaning age and body weight were 28 days and 6.73 kg, respectively. All fecal samples were collected on the same day to minimize potential variation associated with sampling time. All selected piglets were clinically healthy and had not received therapeutic treatment for disease before sampling. Ear tissue and fecal samples were immediately frozen in liquid nitrogen and stored at −80 °C for further analysis.

### 2.2. DNA Extraction and PCR-RFLP Genotyping of the FUT1 Gene

Genomic DNA was extracted from ear tissue using the genomic DNA extraction kit (BEIJING TIANGEN BIOTECH Co., Ltd., Beijing, China), following the manufacturer’s instructions. The quality and concentration of the extracted DNA were assessed using 1% agarose gel electrophoresis and the EPOCH2 microplate reader (BioTek Instruments, Inc., Winooski, VT, USA). Primers for amplifying the *FUT1* gene M307 locus were cited from Bosworth [17] with sequences F1: 5′-CCAACGCCTCCGATTCCTGT-3′ and F2: 5′-GTGCATGGCAGGCTGGATGA-3′, synthesized by Shanghai Sangon Biotech Co., Ltd. The PCR product was 161bp (Supplementary Figure S1A). The amplified fragments containing the M307 site were digested with FastDigest *Hin*6I, and the products were separated on a 2% agarose gel (Supplementary Figure S1B). Three individuals were randomly selected from each genotype for validation by Sanger sequencing (Shanghai Sangon Biotech Co., Ltd., Shanghai, China).

### 2.3. 16S rRNA Amplicon Sequencing

Fecal genomic DNA was extracted using the E.Z.N.A. Soil DNA Kit (Omega Bio-tek, Inc., Norcross, GA, USA) according to the manufacturer’s protocol. The sample was transferred to a Disruptor Tube containing 725 μL of SLX-Mlus Buffer and vortexed at maximum speed for 3–5 min. After brief centrifugation, 72 μL of DS Buffer was added, and the mixture was incubated at 70 °C for 10 min, followed by centrifugation at 10,000× *g* for 5 min. A 400 μL aliquot of the supernatant was mixed with 135 μL of chilled P2 Buffer and 200 μL of fully resuspended cHTR Reagent and centrifuged at maximum speed for 1 min to remove inhibitors. The cleared supernatant was mixed with an equal volume of XP1 Buffer and loaded onto a HiBind^®^ DNA Mini Column, supplied as part of the E.Z.N.A. Soil DNA Kit. The column was washed with 500 μL of HBC Buffer and twice with 700 μL of DNA Wash Buffer. After centrifugation at maximum speed for 2 min to remove residual ethanol, DNA was eluted with 50 μL of Elution Buffer preheated to 70 °C. The eluate was reapplied to the same column and centrifuged again to improve DNA recovery [18]. The concentration and quality of the genomic DNA were determined by 1.0% agarose gel electrophoresis and the NanoDrop 2000 spectrophotometer (Thermo Scientific, Waltham, MA, USA). DNA samples were stored at −80 °C prior to further use.

The V4 hypervariable region of the bacterial 16S rRNA gene was amplified by PCR using the primer pair 515F (5′-GTGCCAGCMGCCGCGGTAA-3′) and 806R (5′-GGACTACHVGGGTWTCTAAT-3′). Sample specific index sequences were incorporated during library preparation to enable multiplexed sequencing and subsequent demultiplexing. The PCR amplification was carried out under the following conditions: initial denaturation at 94 °C for 5 min, followed by 30 cycles of denaturing at 94 °C for 30 s, annealing at 50 °C for 30 s, and extension at 72 °C for 60 s; a final extension at 72 °C for 7 min. Each sample was amplified in triplicate, and the PCR products were pooled before purification. PCR products were detected by 1.0% agarose gel electrophoresis to confirm the presence and size of the target amplicon (Supplementary Figure S2), then purified using the Agencourt AMPure XP Kit (Beckman Coulter, Inc., Brea, CA, USA). High-throughput sequencing was performed using the Illumina Nextseq2000 (Illumina, Inc., San Diego, CA, USA) platform, following the standard protocols provided by Beijing Allwegene Tech Co., Ltd. (Beijing, China). The results were stored in Fastq format. All samples were processed and sequenced in a single sequencing batch. Therefore, the sequencing batch had no between-batch variations and was not included as a model term.

### 2.4. Data Sequence Processing and Analysis

After demultiplexing, the resulting sequences were quality-filtered with fastp (v0.19.6) [19], and merged with FLASH (v1.2.11) [20]. The raw reads were quality-filtered according to the following criteria: (1) bases with quality scores below 20 at the 3′ end of each read were trimmed using a 10 bp sliding window. When the average quality score within a window fell below 20, the read was truncated from the beginning of that window. Reads shorter than 50 bp after quality filtering and reads containing ambiguous bases (N) were discarded; (2) paired-end reads were merged into a single sequence based on their overlapping regions, with a minimum overlap length of 10 bp; (3) the maximum mismatch ratio permitted within the overlapping region was 0.2, and sequences failing to meet this criterion were discarded; and (4) samples were distinguished according to the barcode and primer sequences at both ends of the reads, and sequence orientations were corrected accordingly. No mismatches were permitted in the barcode sequences, whereas a maximum of two mismatches was allowed in the primer sequences.

Using the default parameters, the quality-filtered and merged sequences, as single end sequences, were subsequently denoised using the DADA2 [21] plugin implemented in the QIIME 2 [22] pipeline (version 2025.4), which infers amplicon sequence variants (ASVs) with single-nucleotide resolution based on sample-specific error profiles. The resulting ASVs were used for taxonomic annotation, diversity analyses, microbial community composition analysis, and differential abundance analysis. Alpha-diversity indices were calculated after rarefying all samples to 40,000 reads using a fixed random seed. The minimum library size was 42,120 reads; therefore, all 163 samples were retained. For beta-diversity analyses, the unrarefied ASV table was converted to within-sample relative abundance before calculating Bray–Curtis dissimilarities.

Taxonomy was assigned in DADA2 against the SILVA nr99 release 138.2 species-aware training set using the naive Bayesian classifier (assignTaxonomy) with a minimum bootstrap confidence of 60. The complete DADA2-formatted SILVA training set was used without primer-region extraction. Because this classifier is bootstrap based, no percent-identity threshold was applied. ASVs unresolved at the genus level were retained in ASV-level diversity analyses but excluded from named-genus summaries and genus-level statistical tests. Functional profiles of the microbial communities were predicted using PICRUSt2 (Phylogenetic Investigation of Communities by Reconstruction of Unobserved States, v2.6.3) [23] based on the representative ASV sequences.

### 2.5. Statistical Analysis

Based on the ASVs information, rarefaction curves and alpha diversity indices including Chao1 richness and the Shannon and Simpson indices were calculated with Mothur (v1.30.1) [24]. In the beta diversity analysis, the similarities among the microbiota of the three genotypes and three pig breeds were determined by principal coordinate analysis (PCoA) based on Bray–Curtis dissimilarity using the Vegan (v2.5-3) package.

Associations of breed and *FUT1* genotype with microbial community composition were evaluated using a marginal permutational multivariate analysis of variance (PERMANOVA) model with 9999 permutations, allowing the contribution of each factor to be assessed while accounting for the other model term. Because the *FUT1* AA genotype occurred only in Duroc animals, an additional genotype-based PERMANOVA was performed using the Duroc subset. Homogeneity of multivariate dispersion was evaluated separately for breed and *FUT1* genotype using bias-adjusted distances of individual samples to their corresponding group centroids, with significance assessed using 9999 permutations; genotype dispersion was additionally examined within Duroc animals. Where applicable, *p* values were adjusted for multiple testing using the Benjamini–Hochberg false-discovery-rate procedure. PERMANOVA and multivariate-dispersion analyses were implemented using the adonis2, betadisper, and permutest functions.

Linear discriminant analysis (LDA) effect size (LEfSe) [25] was performed to identify differentially abundant bacterial taxa, from the phylum to genus levels, among the different groups (LDA score > 2.0 and *p* < 0.05). Statistical differences among groups were assessed using the Kruskal–Wallis test for multiple comparisons and the Wilcoxon rank-sum test for pairwise comparisons, with *p* < 0.05 considered statistically significant.

## 3. Results

### 3.1. Genotyping of FUT1 Gene and ASVs Data Statistics

PCR-RFLP analysis of the M307 locus in the *FUT1* gene among 163 pigs revealed three distinct genotypes: *AA*, *GA*, and *GG*. Notably, the resistant genotype *AA* was observed only in Duroc. The detailed distribution of these genotypes is presented in Table 1.

A total of 13,892,644 raw tags were generated from the high-throughput sequencing of the 163 piglets. Following quality filtering, 13,212,301 clean tags were retained for downstream analysis. Rarefaction curves for most individual samples gradually approached plateaus (Supplementary Figure S3), indicating that the sequencing depth was generally adequate to capture the majority of bacterial ASVs present in the fecal samples.

**Figure 1:**
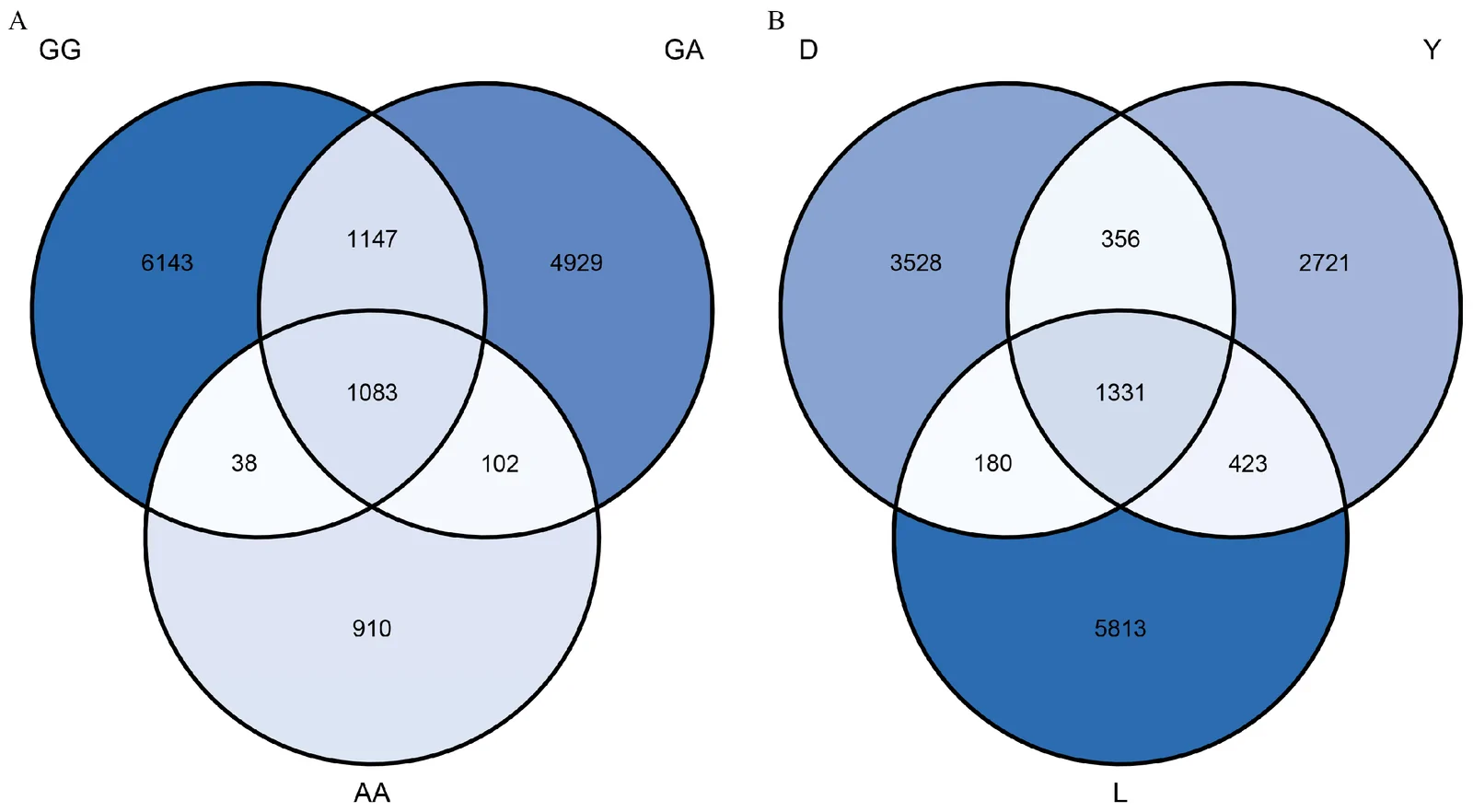
Venn diagrams of gut microbial ASVs across *FUT1* genotypes and pig breeds. (**A**) Shared and unique ASVs among the *GG*, *GA*, and *AA* genotypes. (**B**) Shared and unique ASVs among Duroc (D), Yorkshire (Y), and Landrace (L) pigs. The values indicate the numbers of unique or shared ASVs in the corresponding regions.

Additionally, a total of 14,352 ASVs were identified using the DADA2 pipeline and were subsequently used for downstream analyses. A total of 5395, 7747, and 4831 ASVs were identified in Duroc, Landrace, and Yorkshire, respectively, with 3528, 5831, and 2721 ASVs unique to each breed and 1331 ASVs shared among all three breeds. Similarly, 2133, 7261, and 8411 ASVs were identified in the *AA*, *GA*, and *GG* genotypes, respectively, of which 910, 4929, and 6143 were genotype-specific, while 1083 ASVs were common to all three genotypes (Figure 1). The Venn diagram was drawn using our self-developed ggVenn Diagram software package (v1.5.0) [26].

### 3.2. Analysis of α-Diversity and β-Diversity

Among *α*-diversity indices, including the Chao1, Shannon, and Simpson indices, with the Simpson index calculated as 1 − D, the microbiome diversity of Landrace was highest, followed by Yorkshire and Duroc (Table 2). These three indices differed significantly among the three pig breeds. Specifically, Landrace exhibited the highest Chao1 index, compared to Yorkshire (*p* < 0.01) and Duroc (*p* < 0.001). Regarding the Shannon index, no significant difference was observed between Landrace and Yorkshire. However, both Landrace and Yorkshire exhibited significantly higher Shannon indices compared to Duroc (*p* < 0.001). Regarding the Simpson index, Yorkshire showed a higher value than Landrace pigs, although the difference was not statistically significant. In contrast, the Simpson index was significantly higher in Yorkshire than in Duroc pigs.

The *α*-diversity indices of the *FUT1* genotypes exhibited a different pattern from those observed among the pig breeds (Table 2). Significant differences were detected in the Chao1 and Shannon indices among the three *FUT1* genotypes, whereas no significant difference was observed in the Simpson index. Specifically, the *GA* genotype showed the highest Chao1 index, which was significantly higher than those of the *AA* and *GG* genotypes, while the *AA* genotype exhibited the lowest Chao1 index. In addition, the *GG* genotype had the highest Shannon index, which was significantly higher than those of the *AA* and *GA* genotypes, whereas the *AA* genotype showed the lowest Shannon index.

**Figure 2:**
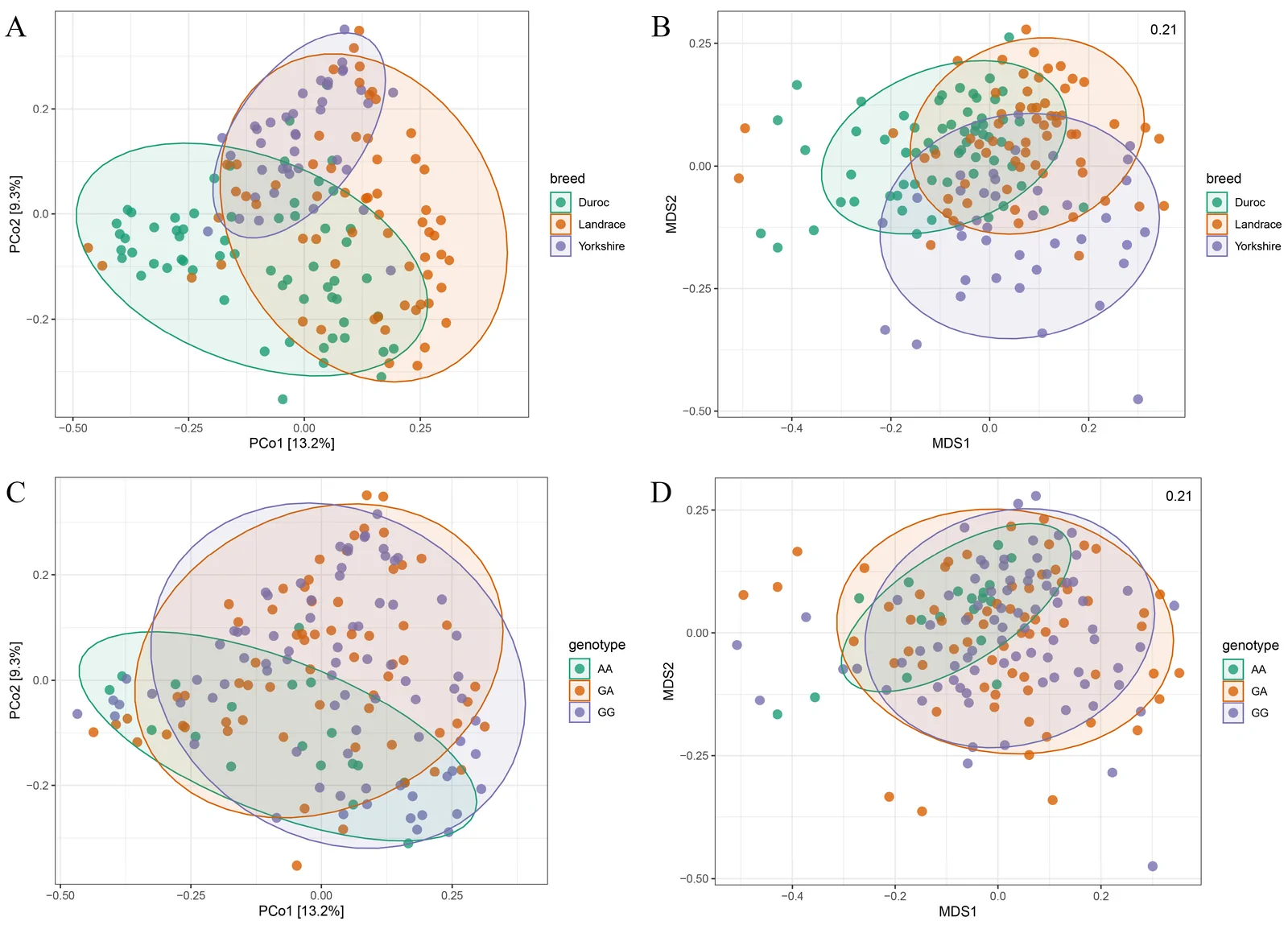
PCoA and NMDS analysis of Bray–Curtis distance matrix. (**A**,**B**) PCoA and NMDS plots of different pig breeds; (**C**,**D**) PCoA and NMDS plots of different genotypes. Each data point represents the clustering pattern of individual samples. The spatial proximity between samples reflects the similarity in their microbial community composition. The closer the distance, the higher the similarity.

The *β*-diversity analysis based on Bray–Curtis dissimilarities revealed partial differences in fecal microbial community structure among pig breeds and *FUT1* genotypes, although substantial overlap was observed among groups (Figure 2). In the breed-based PCoA, the first two principal coordinates explained 13.2% and 9.3% of the total variation, respectively. The NMDS ordination revealed a pattern consistent with that observed in the PCoA, with substantial overlap among the group ellipses indicating only partial differentiation of microbial community structure among breeds. In the genotype-based analyses, the *AA*, *GA*, and *GG* groups also showed extensive overlap in both the PCoA and NMDS plots.

Bray–Curtis-based PERMANOVA showed that breed was significantly associated with microbial community composition, explaining 6.81% of the total variation (R^2^ = 0.0681, pseudo-F = 5.915, BH-adjusted P = 0.0002). After accounting for breed, *FUT1* genotype explained only 1.25% of the variation and was not significantly associated with community composition (R^2^ = 0.0125, pseudo-F = 1.087, BH-adjusted P = 0.2837). Because the AA genotype was observed exclusively in Duroc animals, a sensitivity analysis was conducted within the Duroc population; *FUT1* genotype remained nonsignificant (*n* = 61, R^2^ = 0.0374, pseudo-F = 1.126, P = 0.2538). Analysis of multivariate dispersion indicated significant differences in within-group variability among breeds (F_2,160_ = 5.562, BH-adjusted P = 0.0123), suggesting that the significant breed effect detected by PERMANOVA may reflect both differences in group centroids and differences in dispersion and should therefore be interpreted cautiously. In contrast, genotype-associated dispersion was not significant after multiple-testing correction in either the complete dataset (F_2,160_ = 3.004, raw P = 0.0498, BH-adjusted P = 0.0747) or the Duroc-only subset (F_2,58_ = 0.187, BH-adjusted P = 0.8310).

### 3.3. Gut Microbiome Composition of Different Groups

**Figure 3:**
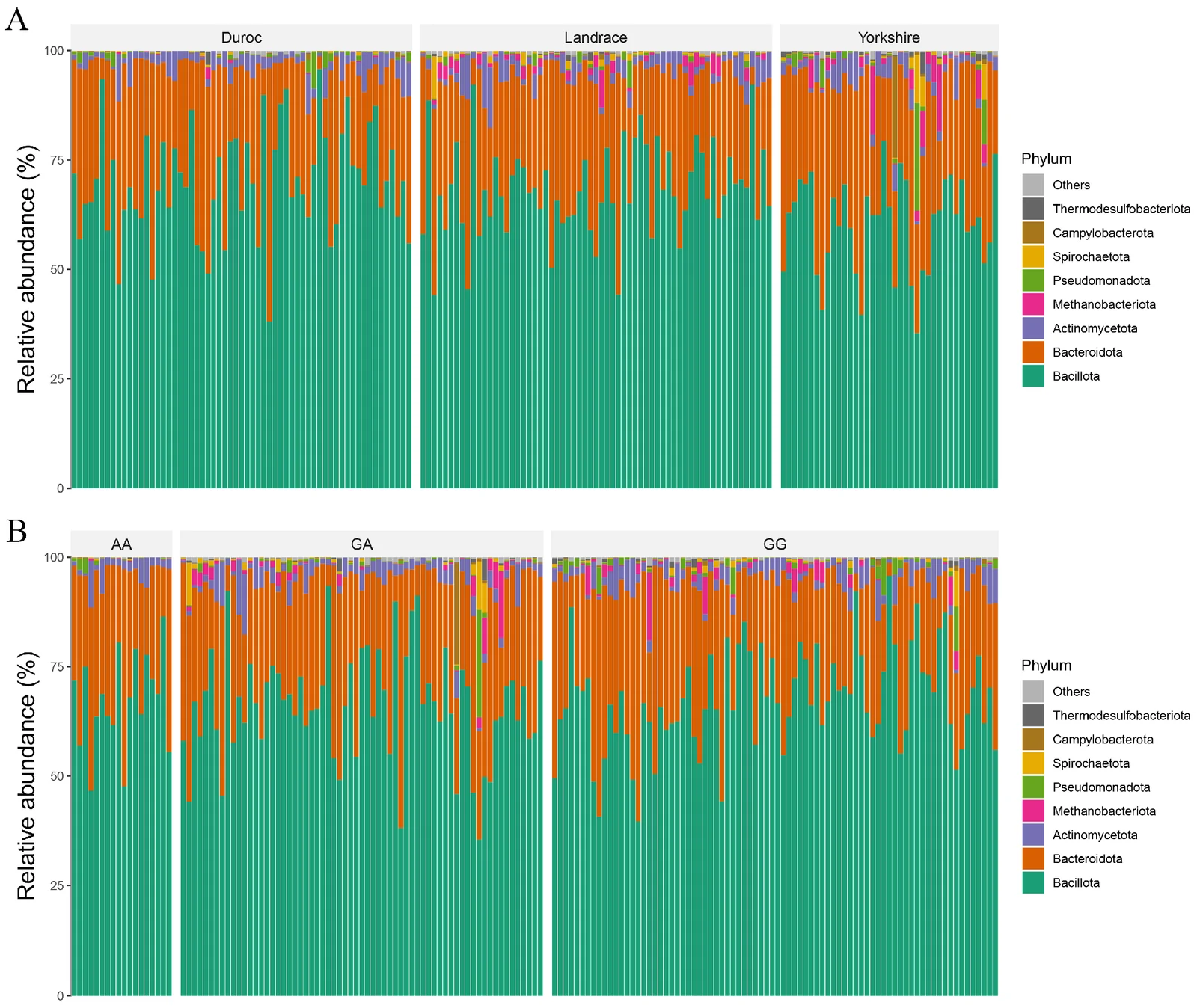
Phylum-level microbiota composition in different pig breeds and *FUT1* genotypes. (**A**) Samples are grouped into Duroc, Landrace, and Yorkshire pigs. (**B**) Samples are grouped into *AA*, *GA*, and *GG* genotypes. The *x*-axis represents individual samples, and the *y*-axis represents the relative abundance (%) of bacterial phyla. Different colors represent different bacterial phyla. These stacked-bar plots are intended to provide a descriptive overview of overall taxonomic composition and inter-individual variation and are not intended for formal statistical comparison of individual phyla among groups.

**Figure 4:**
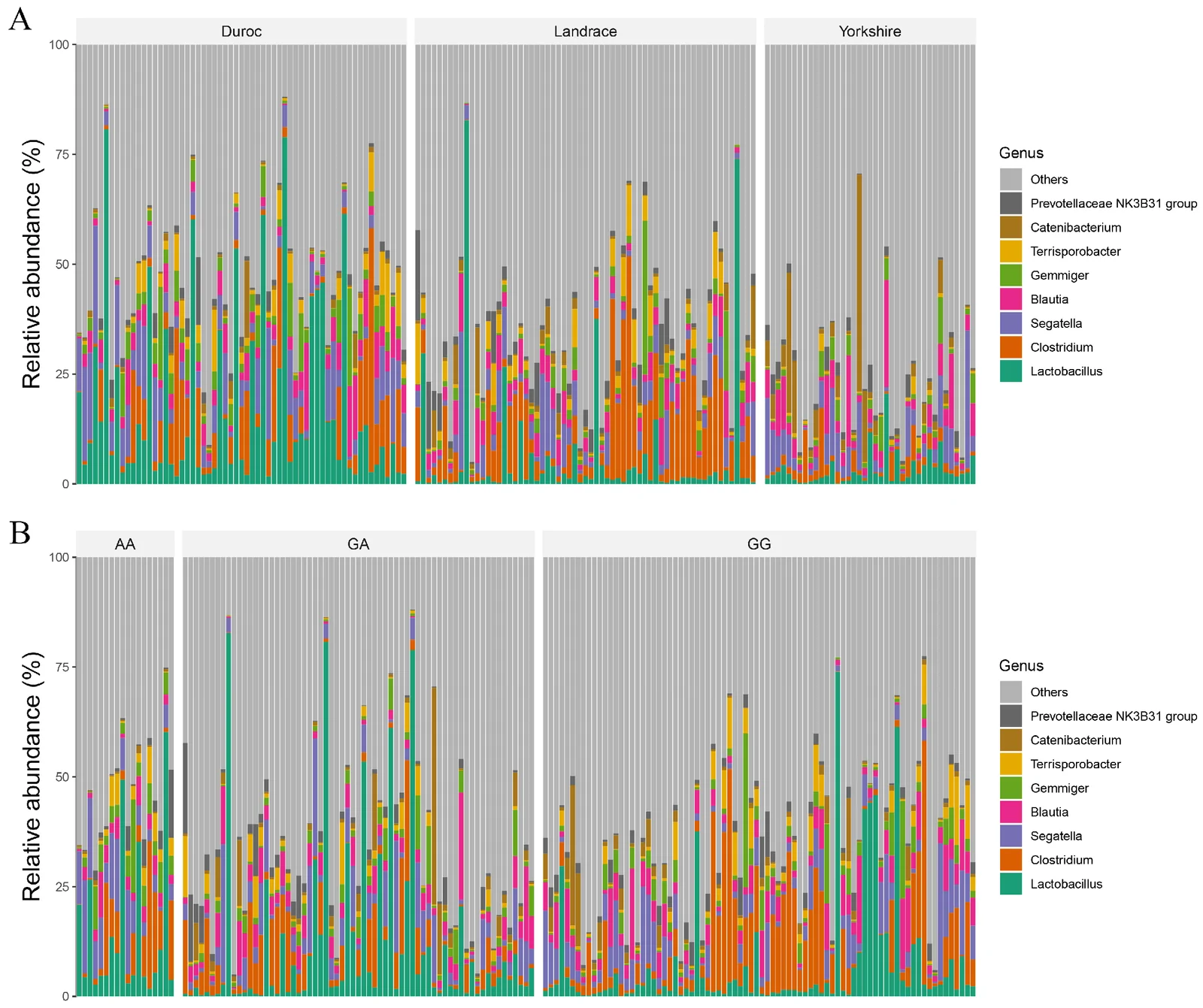
Genus-level microbiota composition in different pig breeds and *FUT1* genotypes. (**A**) Samples are grouped into Duroc, Landrace, and Yorkshire pigs. (**B**) Samples are grouped into *AA*, *GA*, and *GG* genotypes. The *x*-axis represents individual samples, and the *y*-axis represents the relative abundance (%) of bacterial genera. Different colors represent different bacterial genera. These stacked bar plots are intended to provide a descriptive overview of overall taxonomic composition and inter-individual variation and are not intended for formal statistical comparison of individual genera among groups.

ASV-based taxonomic analysis at the phylum and genus level revealed differences in the composition of the gut microbiota among different pig breeds (Figure 3A) and *FUT1* genotypes (Figure 3B). Across all samples, the phyla with the highest mean relative abundances were: *Bacillota*, *Bacteroidota*, *Actinomycetota*, *Methanobacteriota*, *Pseudomonadota*, *Spirochaetota*, *Campylobacterota*, *Thermodesulfobacteriota*, with *Bacillota* and *Bacteroidota* exhibiting the highest abundances. At the genus level, the three pig breeds (Figure 4A) and three genotypes (Figure 4B) exhibited similar microbial profiles, with the commonly abundant genera consisting of *Lactobacillus*, *Clostridium*, *Segatella*, *Blautia*, *Gemmiger*, *Terrisporobacter*, *Catenibacterium*, *Prevotellaceae NK3B31 group*, and others.

**Figure 5:**
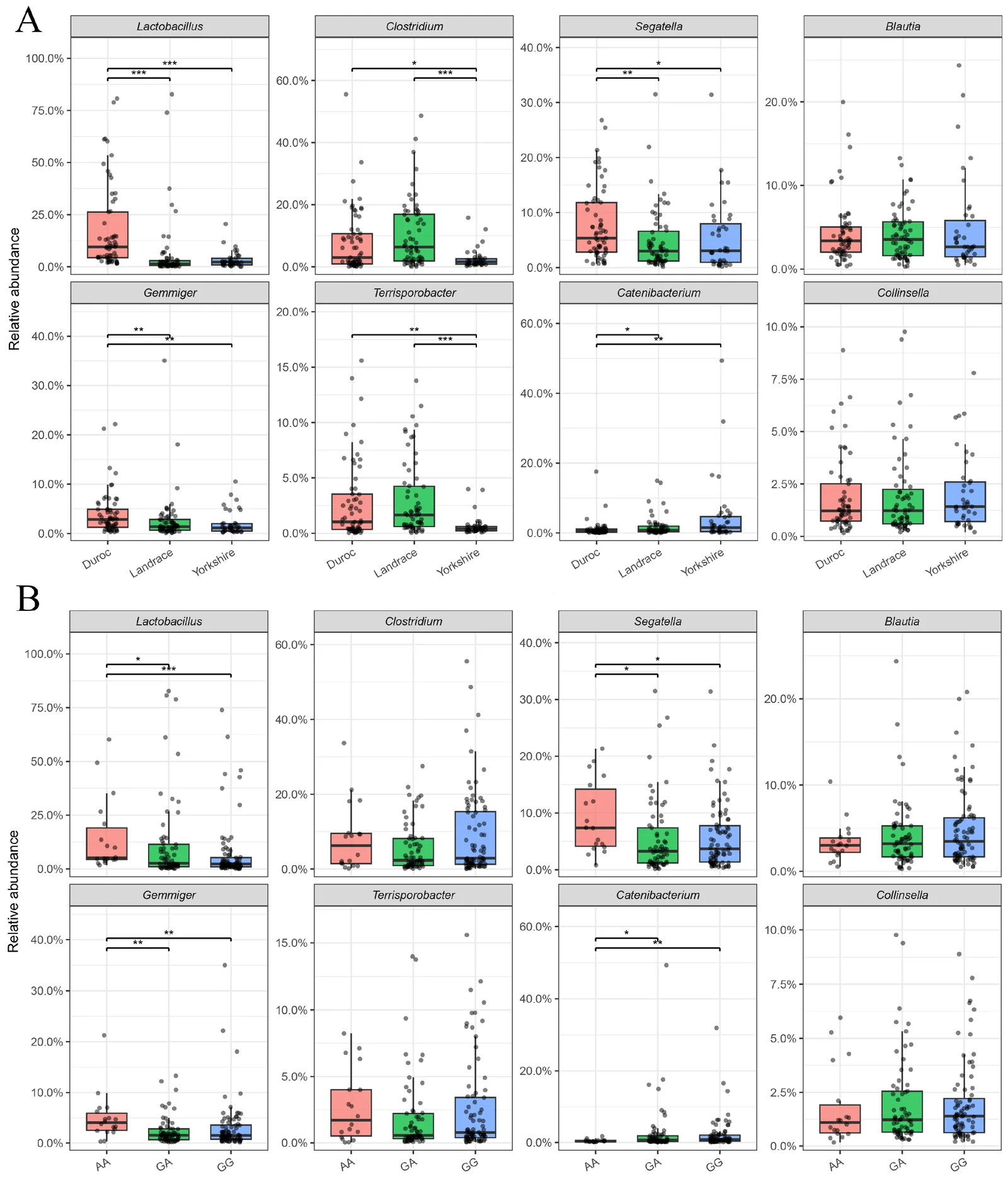
Distribution of selected bacterial genera among different pig breeds and *FUT1* genotypes. (**A**) Boxplots show the relative abundances of top eight bacterial genera in Duroc, Landrace, and Yorkshire pigs. (**B**) Boxplots show the relative abundances of top eight bacterial genera in *AA*, *GA*, and *GG* genotypes. The center line indicates the median, the box represents the interquartile range (IQR), and the whiskers indicate 1.5 × IQR. Each dot represents an individual sample. Different colors represent different pig breeds. * *p* < 0.05; ** *p* < 0.01; *** *p* < 0.001.

Further analysis of the eight most abundant genera revealed marked differences in microbial community composition among Duroc, Landrace, and Yorkshire pigs (Figure 5A). Duroc pigs exhibited a significantly higher relative abundance of *Lactobacillus* than both Landrace and Yorkshire pigs (*p* < 0.001). The relative abundance of *Segatella* was also higher in Duroc pigs than in Landrace (*p* < 0.01) and Yorkshire pigs (*p* < 0.05). Similarly, *Gemmiger* was more abundant in Duroc pigs than in the other two breeds (*p* < 0.01). In contrast, Yorkshire pigs showed lower relative abundances of *Clostridium* than Duroc (*p* < 0.05) and Landrace pigs (*p* < 0.001), as well as lower relative abundances of *Terrisporobacter* than Duroc (*p* < 0.01) and Landrace pigs (*p* < 0.001). The relative abundance of *Catenibacterium* was significantly lower in Duroc pigs than in Landrace (*p* < 0.05) and Yorkshire pigs (*p* < 0.01). No significant breed-associated differences were observed for *Blautia* or *Collinsella*.

Furthermore, genotype-related differences were also evident (Figure 5B). The relative abundance of *Lactobacillus* was significantly higher in individuals with the *AA* genotype than in those with the *GA* (*p* < 0.05) and *GG* genotypes (*p* < 0.001). Similarly, *Segatella* was more abundant in the *AA* group than in both the *GA* and *GG* groups (*p* < 0.05), whereas *Gemmiger* showed significantly higher relative abundance in the *AA* group than in the *GA* and *GG* groups (*p* < 0.01). In contrast, the relative abundance of *Catenibacterium* was significantly lower in the *AA* group than in the *GA* (*p* < 0.05) and *GG* groups (*p* < 0.01).

### 3.4. Taxonomic Profiles of Microbiome

**Figure 6:**
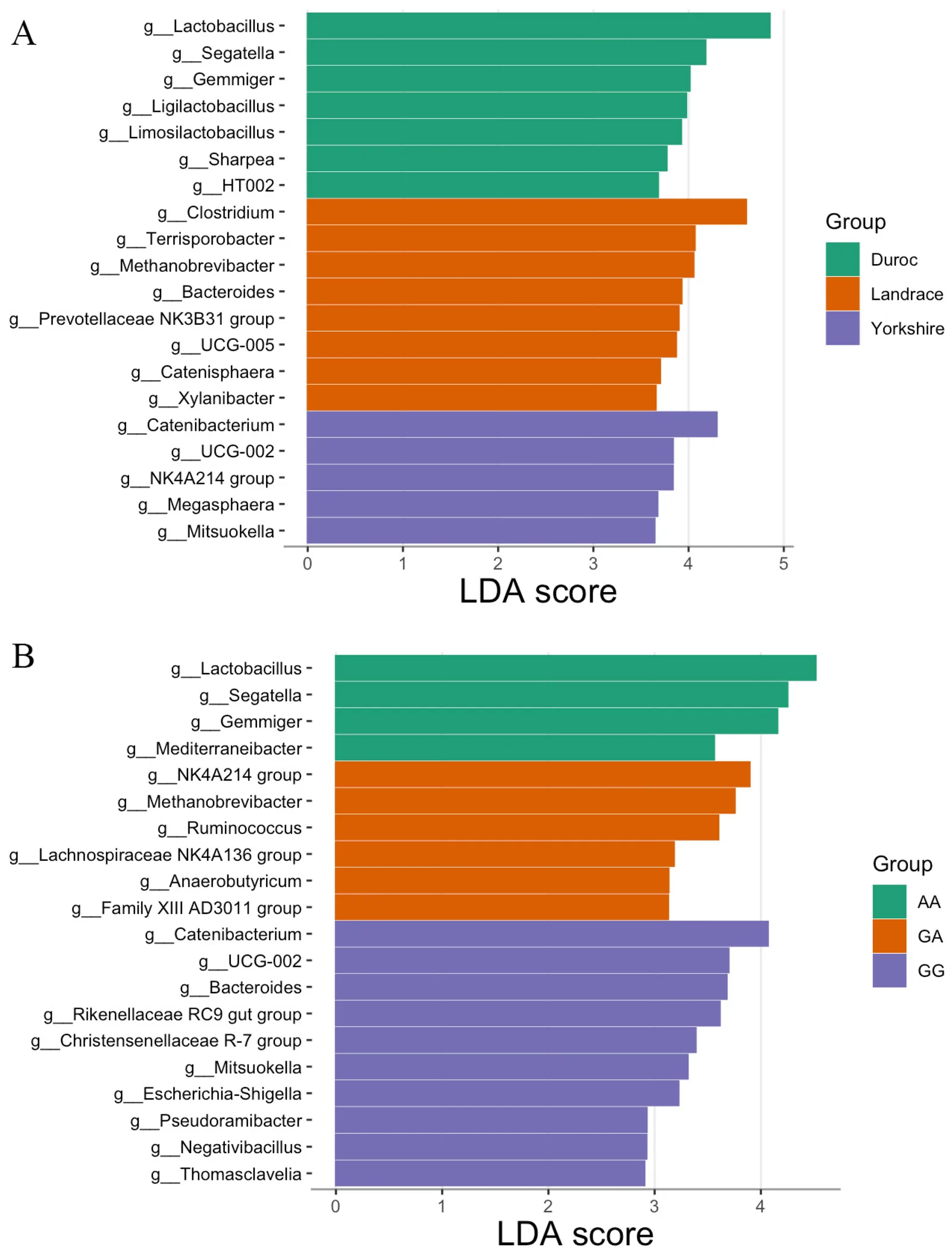
LEfSe analysis of differentially abundant bacterial genera among different pig breeds and FUT1 genotypes. (**A**) Samples are grouped into Duroc, Landrace, and Yorkshire pigs. (**B**) Samples are grouped into *AA*, *GA*, and *GG* genotypes. Bar length represents the LDA score, and bar color indicates the pig breed or genotype in which each genus was significantly enriched.

LEfSe analysis revealed distinct genus-level microbial signatures associated with both breeds and *FUT1* genotypes. Among the three breeds (Figure 6A), seven genera were enriched in Duroc pigs, with Lactobacillus showing the highest discriminatory effect, followed by *Segatella*, *Gemmiger*, *Ligilactobacillus*, *Limosilactobacillus*, *Sharpea*, and *HT002*. Landrace pigs were characterized by the enrichment of eight genera, including *Clostridium*, *Terrisporobacter*, *Methanobrevibacter*, *Bacteroides*, *Prevotellaceae NK3B31 group*, *UCG-005*, *Catenisphaera*, and *Xylanibacter*, of which *Clostridium* had the highest LDA score. In Yorkshire pigs, *Catenibacterium* was the most prominent discriminative genus, together with *UCG-002*, *NK4A214 group*, *Megasphaera*, and *Mitsuokella*. Distinct microbial biomarkers were also observed among the *FUT1* genotypes (Figure 6B). The *AA* genotype was characterized by *Lactobacillus*, *Segatella*, *Gemmiger*, and *Mediterraneibacter*, whereas the *GA* genotype was associated with *NK4A214 group*, *Methanobrevibacter*, *Ruminococcus*, *Lachnospiraceae NK4A136 group*, *Anaerobutyricum*, and *Family XIII AD3011 group*. In contrast, the *GG* genotype exhibited the largest number of discriminative genera, including *Catenibacterium*, *UCG-002*, *Bacteroides*, *Rikenellaceae RC9 gut group*, *Christensenellaceae R-7 group*, *Mitsuokella*, *Escherichia–Shigella*, *Pseudoramibacter*, *Negativibacillus*, and *Thomasclavelia*. Among these taxa, *Lactobacillus*, *Clostridium*, and *Catenibacterium* represented the strongest microbial discriminators of Duroc, Landrace, and Yorkshire pigs, respectively, while *Lactobacillus* and *Catenibacterium* were the leading biomarkers of the *AA* and *GG* genotypes, respectively.

### 3.5. Functional Prediction Analysis of FUT1 Gene Polymorphisms

**Figure 7:**
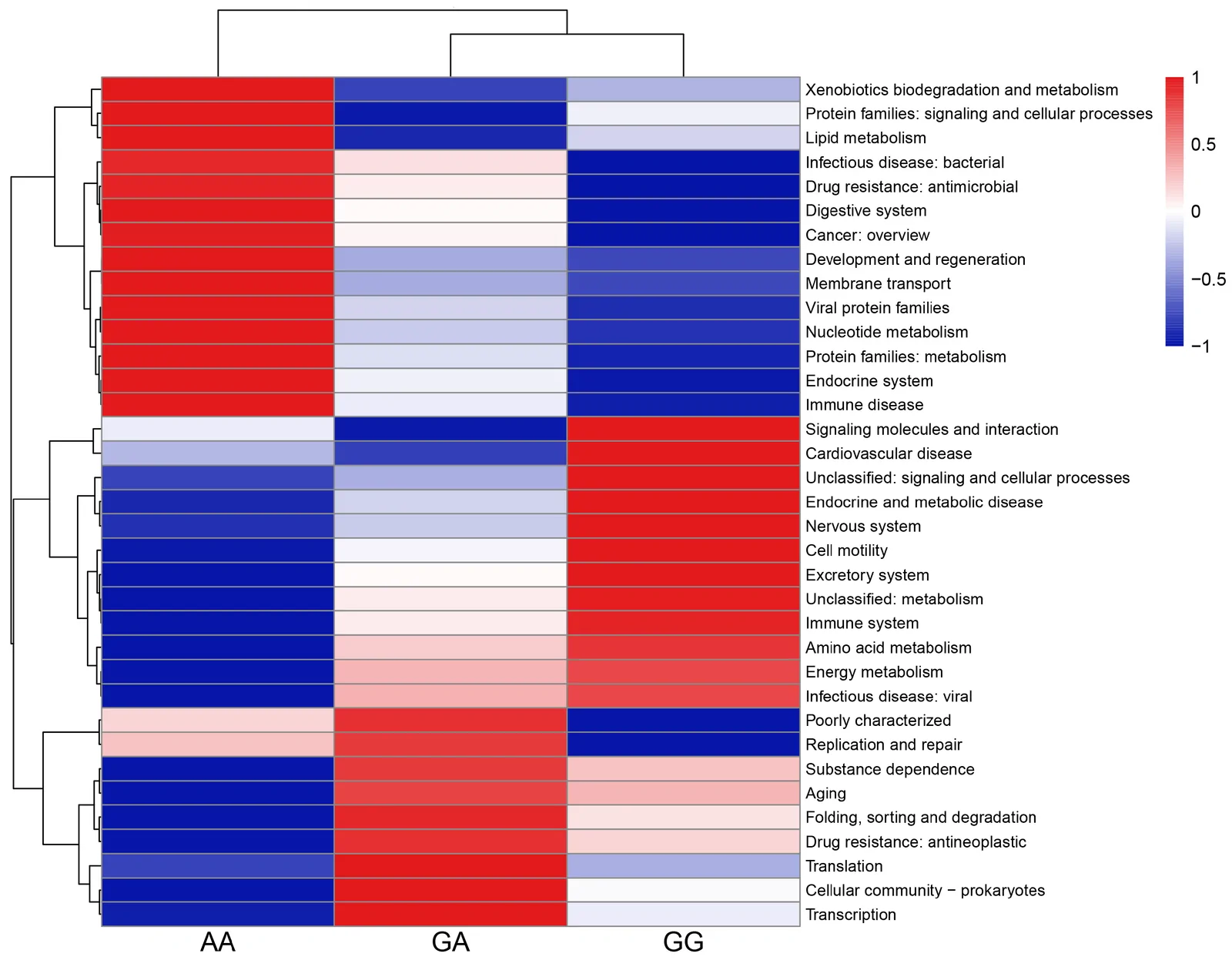
Heatmap of KEGG pathways for different genotypes. Rows represent broad KEGG level-2 functional categories assigned to predicted microbial gene families, and columns represent the mean predicted functional profiles of the *AA*, *GA*, and *GG* genotype groups. Colors indicate row-wise standardized values, with red and blue denoting relatively higher and lower predicted representation, respectively. These values represent predicted microbial functional potential rather than directly measured microbial activity. KEGG labels such as “Immune system”, “Digestive system”, and “Endocrine system” are broad database annotation categories and do not represent measurements of host immune, digestive, or endocrine physiology.

To examine the functional profiles of the gut microbiome across different genotypes, we performed functional prediction and annotation using PICRUSt2 based on the KEGG database. The results showed that the *GA* and *GG* groups were more similar to each other, whereas the *AA* group formed a separate branch, indicating a relatively distinct predicted functional configuration in the *AA* genotype (Figure 7). Among the pathways displayed in the heatmap, the *AA* group showed higher predicted representation of microbial gene families assigned to xenobiotics biodegradation and metabolism, lipid and nucleotide metabolism, membrane transport, and protein families involved in metabolism and cellular signaling. The *AA* group also showed higher predicted representation of microbial gene family annotations assigned to broad KEGG level-2 categories labeled “Digestive system” and “Endocrine system”. In contrast, the *GG* group was characterized by higher predicted representation of amino acid and energy metabolism, cell motility, signaling molecules and interaction, unclassified metabolic pathways, and microbial gene families assigned to the KEGG category labeled “Immune system”. The *GA* group exhibited comparatively higher predicted abundances of pathways related to genetic information processing and protein homeostasis, including transcription, translation, replication and repair, and protein folding, sorting, and degradation. These KEGG category names represent database annotations of predicted microbial gene families and should not be interpreted as measurements of host immune, digestive, or endocrine activity. Collectively, these results indicate genotype-associated differences in the predicted functional potential of the gut microbiota, with the *AA*, *GA*, and *GG* genotypes displaying functional patterns primarily related to xenobiotic and lipid processing, genetic information processing, and energy and amino acid metabolism, respectively.

## 4. Discussion

### 4.1. FUT1 Genotype, Breed, and Microbial Diversity

The *FUT1* gene is associated with disease resistance in pigs, and the resistant *AA* genotype has been linked to improved growth performance [9] and survival rates in piglets [6]. However, the impact of *FUT1* gene polymorphisms on the composition of the gut microbiome in pigs remains unexplored. In this study, 16S rRNA sequencing technology was employed to analyze fecal samples from 163 pigs across three breeds, aiming to determine whether microbial community structure differed according to breed and *FUT1* genotype.

In the *α*-diversity analysis, significant differences in microbiome diversity were observed among the pig breeds. Landrace pigs exhibited the highest microbial diversity, followed by Yorkshire and Duroc. Among the genotypes, the *AA* genotype displayed the lowest *α*-diversity, whereas the *GG* genotype showed the highest diversity. This difference may be related to the distribution of genotypes across breeds. *β*-diversity analysis revealed that the distances between samples of the three populations and the three genotype groups were relatively close. These findings suggest that the differences in microbiome composition among the samples were relatively small. It is hypothesized that the uniform breeding environment and management of the selected piglets may have contributed to a relatively similar level of diversity in the gut microbiome across different breeds and genotypes.

### 4.2. Breed- and Genotype-Associated Taxonomic Patterns

At the phylum level, *Bacillota* and *Bacteroidota* were identified as the dominant phyla in the samples, with *Bacillota* exhibiting the highest relative abundance. This finding is consistent with previous porcine microbiome studies [15,27,28]. These bacterial groups are closely associated with metabolic functions and immune regulation [29]. No significant phylum-level differences were detected among breeds or genotypes, which may reflect the uniformly healthy condition of the sampled pigs. At the genus level, Duroc, particularly individuals with the *AA* genotype, exhibited significantly higher abundance of *Lactobacillus* compared to other groups. *Lactobacillus*, an important probiotic in swine production, has been shown to improve growth performance, carcass quality [30], and immune function [31], while also exhibiting antimicrobial activity against common intestinal pathogens [32]. Furthermore, *Segatella* was another microbe highly enriched in Duroc pigs and individuals with the *AA* genotype. *Segatella*, particularly *Segatella copri* formerly classified as *Prevotella copri*, is a prevalent carbohydrate-utilizing member of the porcine gut microbiota and has been associated with weaning adaptation, intestinal barrier function, inflammation, growth performance, and host metabolism [33,34]. Experimental studies have shown that *Prevotella copri* can promote fat accumulation in pigs and, under certain conditions, impair growth, intestinal barrier function, and bile acids metabolism in weaned piglets [35,36]. Conversely, another study found that *Prevotella copri* supplementation reduced diarrhea and oxidative injury through modulation of the gut microbiota and the arachidonic acid–AHR–NRF2 pathway [37]. These findings suggest that the biological significance of *Segatella* is likely dependent on strain identity, microbial dose, developmental stage, and the initial intestinal ecosystem.

Landrace pigs displayed a different microbial configuration characterized by *Clostridium*, *Terrisporobacter*, and *Methanobrevibacter*. As a methanogenic archaeon, *Methanobrevibacter* participates in hydrogen disposal during intestinal fermentation. Its abundance can respond to dietary fiber level in pigs [38], suggesting that part of the Landrace-associated profile may reflect interactions between host background and fermentation substrates.

Yorkshire pigs were mainly characterized by *Catenibacterium*, *Megasphaera*, and *Mitsuokella*. These genera are commonly associated with the post-weaning transition toward a plant-based diet and a more mature fermentative microbiota. Early exposure of piglets to solid feed has been reported to accelerate the appearance of post-weaning-associated taxa, including *Catenibacterium* and *Megasphaera*, and to reduce the severity of acute post-weaning disturbances [39].

### 4.3. Predicted Functional Potential

The predicted functional profiles further suggest that *FUT1*-associated differences in taxonomic composition may be accompanied by differences in the predicted functional potential of the gut microbial. The *AA* group showed higher predicted representation of xenobiotic biodegradation, lipid and nucleotide metabolism, and membrane transport, whereas the *GG* group showed higher predicted representation of amino acid and energy metabolism, cell motility, and microbial gene family annotations assigned to the broad KEGG category labeled “Immune system”. The *GA* group showed a distinct pattern involving transcription, translation, replication and repair, and protein folding and degradation. The KEGG category labels shown here represent database annotations of predicted microbial gene families and should not be interpreted as direct measurements of host physiological activity. Interestingly, the clustering of *GA* and *GG* and the separation of *AA* are consistent with the taxonomic pattern reported by Luise et al. [9] in which *AA* pigs differed from *AG* and *GG* pigs. One possible mechanism is that *FUT1* dependent mucosal fucosylation changes bacterial attachment sites and substrate availability, resulting in the selection of microbial communities with different genomic repertoires. However, the present data cannot establish a causal pathway from *FUT1* polymorphism to microbial function or host phenotype.

PICRUSt2 results represent predicted functional potential rather than measured microbial activity. The method infers gene family content from 16S rRNA profiles and reference genomes; it does not quantify gene expression, protein production, or metabolite flux [23]. Accuracy varies across sample types and functional categories, and recent benchmarking has shown that 16S-based tools may have limited sensitivity for biologically meaningful group differences [40,41]. Accordingly, the predicted pathways should be treated as hypotheses for targeted metagenomic and metabolomic validation.

Overall, gut microbiota composition differed among *FUT1* genotypes across pig breeds. The *AA* genotype was associated with a distinct fecal microbial profile characterized by higher relative abundances of *Lactobacillus*, *Segatella*, and *Gemmiger*. However, the *AA* genotype was detected exclusively in Duroc pigs. Therefore, the pooled genotype analysis was confounded by breed and cannot distinguish genotype-associated differences from breed-associated differences. In addition, no disease resistance phenotype or other direct health or production trait was evaluated. Thus, the biological relevance of the observed microbial associations, particularly their potential relationship with disease resistance, remains to be determined.

## 5. Conclusions

In summary, both pig breed and *FUT1* genotype were associated with variation in the gut microbiome of weaned piglets. *Lactobacillus*, *Segatella*, and *Gemmiger* were enriched in Duroc pigs and were also more abundant in pigs carrying the *AA* genotype. These associations may be relevant to intestinal fermentation, barrier function, bile-acid transformation, and dietary responses, but they remain hypothesis generating because microbial metabolites and host phenotypes were not measured.

## Figures and Tables

**Table 1:** Statistical results of *FUT1* gene polymorphisms in different pig breeds.

Pig Breeds	*n*	Genotyping			Gene Frequency	
		*AA*	*GA*	*GG*	*A*	*G*
Duroc	61	18	23	20	0.484	0.516
Yorkshire	39	0	19	20	0.244	0.756
Landrace	63	0	23	40	0.183	0.817

**Table 2:** Alpha diversity indices of different pig breeds and *FUT1* genotypes.

		Index		
		Chao1	Shannon	Simpson
Breed Groups	Duroc	372.197 ± 96.625	3.744 ± 0.618	0.916 ± 0.077
	Landrace	509.175 ± 128.103	4.247 ± 0.634	0.942 ± 0.072
	Yorkshire	493.769 ± 113.274	4.173 ± 0.458	0.945 ± 0.044
	*p*-value	0.0000	0.0000	0.0018
Genotype Groups	*AA*	358.833 ± 67.824	3.763 ± 0.502	0.920 ± 0.072
	*GA*	466.692 ± 134.179	4.026 ± 0.708	0.927 ± 0.086
	*GG*	465.562 ± 128.881	4.117 ± 0.577	0.941 ± 0.051
	*p*-value	0.0018	0.0199	0.2671

## Data Availability

The 16S rRNA sequencing data generated in this study have been deposited in the NCBI BioProject database under accession number PRJNA1223651 and are freely accessible.

## Supplementary Materials

The following supporting information can be downloaded at: https://www.mdpi.com/article/10.3390/microorganisms14081756/s1, Table S1: Composition and nutrient level of experimental diet (as fed-basis). Figure S1: Agarose gel electrophoresis. Figure S2: Electrophoresis results of the amplification products. Figure S3: Rarefaction curves of fecal microbiota samples.

## Abbreviations

The following abbreviations are used in this manuscript:

ASVs	Amplicon sequence variants
*FUT1*	Fucosyltransferase 1
LDA	Linear discriminant analysis
PCoA	Principal coordinate analysis
PERMANOVA	Permutational multivariate analysis of variance
PICRUSt2	Phylogenetic investigation of communities by reconstruction of unobserved states
