# Supplementary material for: Associations of Breed and FUT1 Genotype with the Fecal Microbiota of Weaned Piglets
Source: Microorganisms. 2026 Aug 10;14(8):1756. doi: 10.3390/microorganisms14081756 (PMC13515847; doi:10.3390/microorganisms14081756)
Supplement: Supplementary file 1 [file microorganisms-14-01756-s001.zip › microorganisms-4469289-supplementary.pdf]

Table S1. Composition and nutrient level of experimental diet (as fed-basis)

Items	Lactating Sow	Litter (creep feed)
Ingredients		
Corn	58.81	23.10
Soybean meal	26.50	14.34
Wheat bran	8.61	0.00
Full-fat soybean meal	0.00	13.08
Whey permeate powder	0.00	20.00
Skim milk powder	0.00	12.50
Soybean oil	2.50	8.50
Barley	0.00	5.00
CaHPO4	1.45	0.00
Mono-dicalcium phosphate	0.00	0.55
Limestone	1.05	0.70
Salt (NaCl)	0.50	0.30
L-Lysine HCl	0.08	0.62
DL-Methionine	0.00	0.36
L-Threonine	0.00	0.37
L-Tryptophan	0.00	0.14
L-Valine	0.00	0.13
Vitamin-mineral premix [1)]	0.50	0.30
Phytase	0.00	0.01
Total	100.00	100.00
Nutrient levels [2)]		
Digestible energy (DE), MJ/kg	13.94	16.20
Dry matter (DM), %	88.04	91.20
Crude protein (CP), %	18.11	18.20
Crude fiber (CF), %	3.13	1.80
Neutral detergent fiber (NDF), %	11.55	NR
Acid detergent fiber (ADF), %	4.78	NR
Total lysine, %	NR	1.62
SID lysine, %	NR	1.53
Calcium (Ca), %	0.80	0.82
Total phosphorus (TP), %	0.68	0.60

Items	Lactating Sow	Litter (creep feed)
Available phosphorus (AP), %	0.34	0.46

1) The sow-diet premix provided the following per kg of diet: vitamin A, 12 000 IU; vitamin E, 24 IU; vitamin K_3, 2.0 mg; thiamine, 2.0 mg; riboflavin, 6.0 mg; pyridoxine, 4.0 mg; vitamin B_{12}, 24 g; niacin, 30 mg; pantothenic acid, 20 mg; folic acid, 3.6 mg; biotin, 0.4 mg; choline chloride, 0.4 mg; Fe, 96 mg; Cu, 8.0 mg; Zn, 120 mg; Mn, 40 mg; I, 0.56 mg; Se, 0.4 mg.

The litter-diet premix provided per kg of complete diet: Cu, 100 mg; Fe, 90 mg; Mn, 47 mg; Zn, 120 mg; I, 0.6 mg; Se, 0.3 mg; vitamin A, 2.1 mg; vitamin D_3, 25 μg; vitamin E, 100 mg; vitamin K_3, 4 mg; vitamin B_{12}, 15 μg; riboflavin, 2 mg; nicotinic acid, 12 mg; pantothenic acid, 10 mg; choline chloride, 250 mg; vitamin B_1, 2 mg; and vitamin B_6, 3 mg. The litter diet contained 1,000 phytase units (FYT)/kg feed.

2) DM, CP, CF, Ca and TP were measured values, while the other nutrient levels were calculated values.

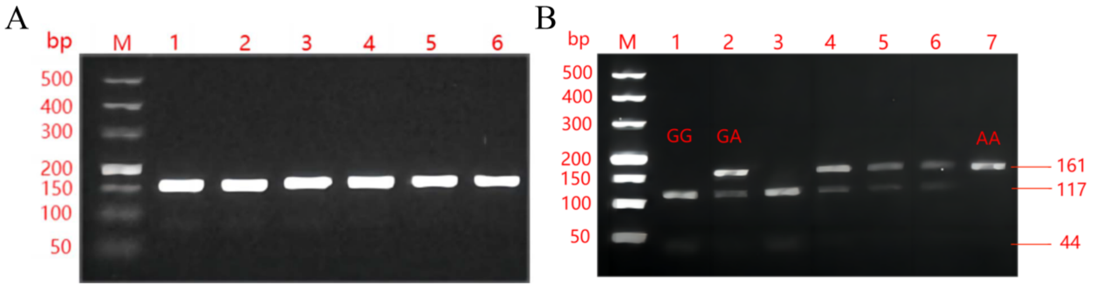

Figure S1. Agarose gel electrophoresis. (A) PCR products of M307 of *FUT-1* gene; (B) PCR-RFLP results of *FUT-1* gene M307 locus. M: 500bp marker

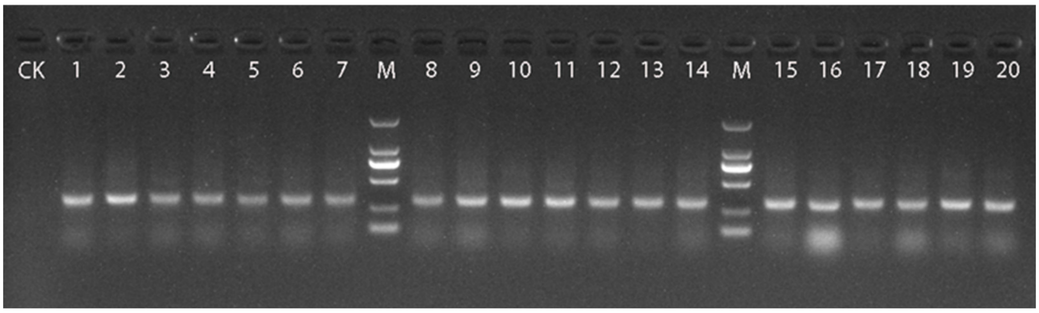

Figure S2. Electrophoresis results of the amplification products. M: DL2000 marker

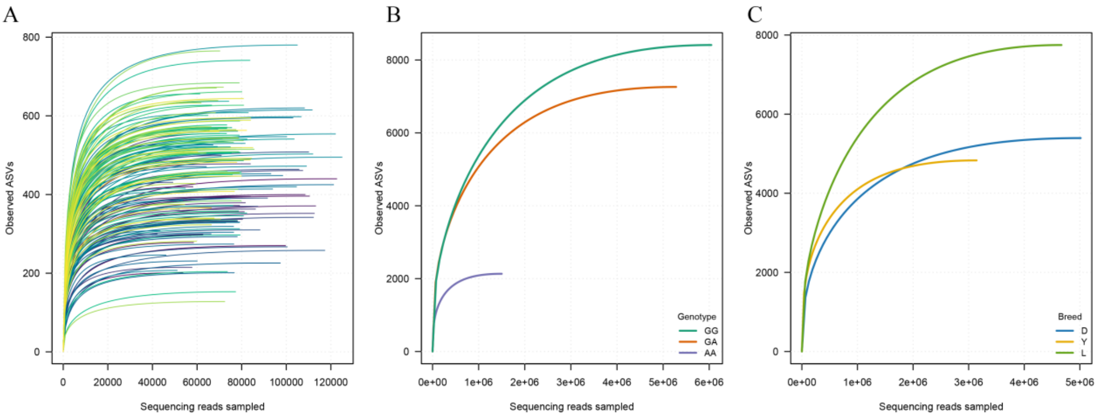

Figure S3. Rarefaction curves of fecal microbiota samples. (A) Rarefaction curves for individual samples; (B) cumulative rarefaction curves grouped by FUT1 genotype, including GG (n=80), GA (n=65), and AA (n=18); and (C) cumulative rarefaction curves grouped by pig breed, including Duroc (n=61), Yorkshire (n=39), and Landrace (n=63). Group level cumulative ASV counts should be interpreted with consideration of differences in sample size and total sequencing effort.
